# Synthetic biology towards the synthesis of custom-made polysaccharides

**DOI:** 10.1111/1751-7915.12241

**Published:** 2015-01-28

**Authors:** Bernd H A Rehm

**Affiliations:** Institute of Fundamental Sciences, Massey UniversityPalmerston North, New Zealand; MacDiarmid Institute for Advanced Materials and NanotechnologyPalmerston North, New Zealand

Polysaccharides and their inherent structural and chemical variability provide an enormous hitherto unexploited design space for production of a range of new biobased materials. Exopolysaccharides are synthesized by numerous microorganisms (Rehm, 2010). Studies on biosynthesis provided critical information on how precursors are enzymatically diverted from primary metabolites and how they are polymerized and secreted. Polysaccharides can be composed of repeating and non-repeating building blocks, which can vary from sugars to sugar acids plus modifications including acetylation and pyruvylation. Building blocks can be single sugars (acids) or multiple sugars (acids). The vast diversity of possible polysaccharides and their corresponding differing material properties offers an almost unlimited source of biobased gel-forming materials with the potential to be made to order. In particular, viscoelastic and biological properties are often critical for the performance of these materials in medical applications such as e.g. tissue engineering, encapsulation of cells, enzymes and/or drugs for controlled and targeted delivery. Structure–function relationships i.e. linking the chemical structure of polysaccharides with materials properties will increasingly inform the *in silico* design of polysaccharides in regard to sugar composition, modifications and glycosidic bond configuration. Ultimately a demand for certain material properties will *in silico* be translated into the chemical structure of the desired polysaccharides. This will then inform the design and assembly of synthetic genes encoding enzymes and proteins for polysaccharide synthesis and secretion. Increasing knowledge about sugar metabolism and hence the possibility to engineer nucleotide sugars or sugar acids biosynthesis routes will enable to provide activated precursors that are ultimately used as building block or part of a building block. Glycosyltransferases catalyse the glycosidic linkage between the sugars/sugar acids. Rational design and engineering of glycosyltransferases will be aligned with the building block design as these transferases will specifically and sequentially incorporate individual sugars/sugar acids, which could be followed by modifications such as acetylation implementing modifying enzymes e.g. acetyltransferases. Such oligosaccharide repeating units will be synthesized on the cytosolic site of the cytoplasmic membrane linked to lipid carrier (bactoprenol, C55). The flippase (Wzx) and polymerase (Wzy) will mediate transfer of the building block across the membrane with subsequent polymerisation. The molecular mechanisms of these processes are currently not fully understood but will likely be elucidated in future for production of custom-made extracellular polysaccharides (Islam and Lam, 2013).

Major advances were achieved elucidating the molecular mechanisms of microbial cellulose (homopolymer of glucose) and alginate (non-repeating sugar acids) synthesis (Morgan *et al*., 2013; Hay *et al*., 2014). In both cases, a membrane-spanning multiprotein complex mediates polymerization and secretion across the cytoplasmic membrane in a very different manner when compared with the synthesis of the Wzy-dependent repeating oligosaccharide polymerization process. Alginate is additionally modified by acetyltransferases, which act while the polymer chain transverses a multiprotein scaffold in the periplasmic space. Alginate itself provides a tremendous design space as it is composed of two different sugar acids (mannuronic acid and guluronic acid) that can randomly alternate or form blocks with the possibility of only mannuronic acid being present. Mannuronic acid can also be acetylated. The molecular weight can likely be controlled by degrading enzymes (lyases) or by engineering the processivity of the polymerase. In addition to the foreseeable *in vivo* engineering of alginate, there is also scope to enzymatically modify alginates isolated from algae and bacteria (Hay *et al*., 2013). The possibility to control the molecular weight, the arrangement of the sugar acids and their acetylation degree will create materials exhibiting a wide range materials and biological properties such as e.g. gel formation and immunogenicity. In general, polysaccharide gels show low elastomeric properties, which could be obtained by blending and/or cross-linking with elastomeric structures. The ability of, in particular, block copolymers to establish intramolecular interactions and morphologies might enable the generation of nano-/micro-structured functionalized materials, which will be driven and controlled by the composition of the polysaccharide (Wen and Oh, 2014).

Another future application of engineering polysaccharide biosynthesis will be the recombinant production of capsular polysaccharides and respective oligosaccharides, which are currently used to produce conjugate vaccines but which are costly isolated from the actual pathogen such as e.g. *Neisseria meningiditis* or *Streptococcus pneumoniae*. This will lower the cost of vaccine production for more extended vaccination programmes enabling better control of infectious diseases.

Access to custom-made polysaccharides will open up a new biomaterials world revolutionizing medical applications (e.g. tissue engineering, drug delivery, wound healing, vaccines and diagnostics) by creating functionalized and responsive material. Functional self-organizing structures will also create opportunities for technical implementation (e.g. enzyme/cell immobilization, bioprocessing, biosensor and research tools).
